# Low‐grade glial neoplasms of germ cell origin may represent maturation of embryonic‐type neuroectodermal elements

**DOI:** 10.1111/his.70007

**Published:** 2025-09-22

**Authors:** João Lobo, Nuno Tiago Tavares, Fernanda Fernandes‐Pontes, Carmen Jerónimo, Rui Henrique, Yiying Yang, Matija Snuderl, Melissa Hruby, Muhammad T Idrees, Thomas M Ulbright, Andres M Acosta

**Affiliations:** ^1^ Department of Pathology Portuguese Oncology Institute of Porto (IPO Porto)/Porto Comprehensive Cancer Center Raquel Seruca (P.CCC) Porto Portugal; ^2^ Cancer Biology and Epigenetics Group, IPO Porto Research Center (GEBC CI‐IPOP), Portuguese Oncology Institute of Porto (IPO Porto)/Porto Comprehensive Cancer Center Raquel Seruca (P.CCC) Porto Portugal; ^3^ Department of Pathology and Molecular Immunology, ICBAS—School of Medicine and Biomedical Sciences University of Porto Porto Portugal; ^4^ Doctoral program, ICBAS—School of Medicine and Biomedical Sciences University of Porto Porto Portugal; ^5^ Department of Pathology, Langone Health New York University New York New York USA; ^6^ Department of Pathology Indiana University Indianapolis Indiana USA

**Keywords:** biomarkers, epigenetics, glial tumours, microRNA‐371‐373, teratoma, testicular germ cell tumours

## Abstract

**Aims:**

Glial tumours of germ cell origin are relatively rare in men, occurring predominantly after chemotherapy. Many exhibit low‐grade histological features within a spectrum that includes teratomas with mature glial/ganglioglial elements and pure low‐grade tumours with glial/ganglioglial phenotype (LGGT) that resemble gliomas/gangliogliomas of the central nervous system. Because foci of glial differentiation are very often seen in association with embryonic‐type neuroectodermal tumour (ENT), we hypothesise that LGGTs may represent differentiation of embryonic‐type neuroectodermal elements of teratoma and/or ENT.

**Methods and results:**

To address this hypothesis, we compared LGGTs, ENT, non‐teratomas, and teratomas using microRNA and DNA methylation analyses. Seven LGGTs underwent microRNA‐371~373 analysis and genomic methylation profiling. Evidence of a prior or concurrent germ cell tumour component containing embryonic neuroectoderm (including overt ENT) was present in 4 LGGTs. None of the tested LGGTs were positive for miR‐371a‐3p, with three cases demonstrating low levels of expression within the so‐called “grey zone”. Unsupervised clustering based on microRNA‐371~373 showed two clusters, one comprising non‐teratomas and another including teratomas, ENTs, and LGGTs. Clustering according to top‐differentially methylated probes did not demonstrate a clear separation according to histology. Genome‐wide assessment of mean methylation levels using violin plots demonstrated that LGGT show a methylation profile “intermediate” between ENT and teratoma.

**Conclusions:**

These results suggest that LGGTs of germ cell origin result from the maturation of ENT components.

## Graphical abstract

Our results support that low grade glial tumours of germ cell origin (LGGTs) represent a phenomenon of maturation (either spontaneous or induced by therapy) of embryonic‐type neuroectoderm, being possibly equivalent to teratoma from a biological perspective.

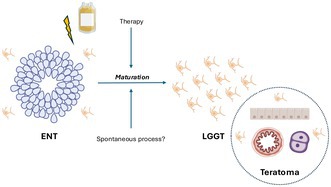

**Abbreviations**
ChT: chemotherapy
EC: embryonal carcinoma
ENT: embryonic‐type neuroectodermal tumour
GCNIS: germ cell neoplasia *in situ*
LGGT: low‐grade glial/ganglioglial tumour of germ cell origin
miR: micro RNA
RMS: rhabdomyosarcoma
RPLND: retroperitoneal lymph node dissection
SM: somatic‐type malignancy of germ cell origin
TGCT: testicular germ cell tumour
YST: yolk sac tumour

## Introduction

Testicular germ cell tumours (TGCTs) represent the most common solid neoplasm of young‐adult men in Western countries, and their incidence is increasing worldwide.^1^ They are broadly categorised into germ cell neoplasia *in situ* (GCNIS)‐derived and GCNIS‐independent, the latter including prepubertal‐type yolk sac tumour and teratoma in children and spermatocytic tumour in adult men.^2^ GCNIS‐derived neoplasms, which comprise the vast majority of TGCTs and originate from reprogrammed primordial germ cell precursors close to the gonocyte stage, are malignant by definition.^3^ These are further divided into seminoma and non‐seminoma, the latter including embryonal carcinoma, postpubertal‐type yolk sac tumour, choriocarcinoma, postpubertal‐type teratoma, and mixed neoplasms containing at least one non‐seminoma component. This classification is clinically relevant because management strategies are different for seminoma and non‐seminoma.^4^, ^5^

The range of post‐chemotherapy induced changes in TGCTs includes the development of so‐called “somatic‐type” malignancies (SMs), characterised by overgrowth of histologic components that resemble a true somatic neoplasm.^6^, ^7^ Although this phenomenon occurs more commonly after systemic therapy, it is also well documented in primary tumours without prior treatment.^8^, ^9^ SMs are largely resistant to systemic therapy, and their occurrence in metastatic sites is associated with poor outcomes if complete resection is not feasible.^6^, ^9^ By contrast, SMs occurring in primary tumours of patients with clinical stage I disease show survival rates similar to those seen in patients with clinical stage I non‐seminoma.^9^, ^10^ Considering these data, patients with clinical stage I SMs could undergo surveillance after orchiectomy. However, given the resistance of these neoplasms to systemic therapy, upfront retroperitoneal lymph node resection is often undertaken with the aim of resecting subclinical metastases that will not respond to platinum‐based chemotherapy.

Historically, SMs were thought to arise as a consequence of “transformation” of elements of teratoma, justifying the current nomenclature endorsed by the World Health Organization classification: Teratoma with somatic‐type malignancy.^11^ Several recent clinicopathologic and molecular studies have provided evidence to support that a subset of SMs arises from non‐teratoma.^8^, ^12^, ^13^, ^14^, ^15^, ^16^, ^17^ The spectrum of neoplasms that meet diagnostic criteria for SM is wide, including primitive (embryonic‐type) tumours, sarcomas, and carcinomas, among others.^10^, ^18^ A particularly intriguing and incompletely uderstood phenotype of TGCT is characterised by low‐grade glial or ganglioglial morphology (LGGT hereafter).^19^, ^20^, ^21^ If these histologic components form a distinct focus larger than 5 mm, they meet diagnostic criteria for SM. In our experience, LGGTs are typically bulky and often represent the predominant or sole neoplastic component of post‐chemotherapy retroperitoneal metastases of TGCTs.

Foci of glial differentiation are often seen in association with embryonic‐type neuroectodermal components present in teratoma and embryonic‐type neuroectodermal tumour (ENT). Therefore, we hypothesise that LGGTs orginate as a consequence of the maturation of embryonic‐type neuroectodermal elements, mostly (albeit not exclusively) after chemotherapy. To assess this hypothesis, we analysed the genomic methylation and microRNA (miR) expression profiles of LGGTs, comparing them with those of other TGCT types, including teratoma and ENT.

## Materials and Methods

### Samples

This work was performed with the approval of the institutional review board of IPO Porto (CES1/018) and Indiana University (protocol #18697, 2023).

A total of 7 LGGTs from 7 individual patients were retrieved from the archives of the Department of Pathology of Indiana University. Cases were centrally reviewed by the senior author, and selected formalin‐fixed paraffin‐embedded (FFPE) sections/blocks were identified for microRNA and DNA methylation analysis. Given that the microRNA expression and DNA methylation profiles of primary TGCTs with conventional histology have been extensively studied,^17^, ^22^ in this work, we focused on the two phenotypes of interest: LGGT and ENT. Paired assessment of metastatic LGGTs and their corresponding primary tumour was not performed due to limited tissue availability. Instead, unpaired GCNIS‐derived TGCTs from different patients were used as comparators (controls) for the experiments. Specifically, microRNA analyses were performed on 7 LGGTs (Table 1), 13 ENTs, and 16 unpaired GCNIS‐derived TGCTs with conventional histology from the Department of Pathology of IPO Porto (3 seminomas, 3 embryonal carcinomas, 4 postpubertal‐type yolk sac tumours, 2 choriocarcinomas, and 4 postpubertal‐type teratomas). Of note, 11 of the 13 ENTs assessed in this study were previously included in a study by Lobo *et al*.^17^ Genomic DNA methylation analysis was performed on 7 LGGTs, 6 ENTs, and 10 postpubertal‐type teratomas.

**Table 1:** Clinicopathological features of the study cohort

	Primary tumour site	Primary tumour histology	Prior malignancy	Prior ChT	LGGT site	Time to LGGT*	Outcome
1	Mediastinum	70% teratoma with low‐grade glioma 30% YST	n/a	No	Mediastinum	n/a (present in primary tumour)	Progressive disease, involving liver and lung
2	Testis	50% seminoma 40% teratoma 5% EC 5% YST	Contralateral pure seminoma	Yes	Thymus and retroperitoneum	7 months	Unknown
3	Testis	80% EC 20% YST	n/a	Yes	Mediastinum	8 months	Unknown
4	Testis	Teratoma with ENT and RMS	n/a	Yes	Pancreas, adrenal gland, mediastinum	2 years and 3 months	NED 15 months post‐op
5	Unknown, mediastinum favoured	80% EC 10% seminoma <5% choriocarcinoma 3% teratoma 2% YST	n/a	Yes	Mesentery and retroperitoneal lymph nodes	8 months	NED 16 months post‐op
6	Testis	50% teratoma 20% ENT 15% EC 10% YST 5% seminoma	n/a	Yes	Mediastinal lymph nodes	~3 years	NED 6 months post‐op
7	Testis	Teratoma with ENT and RMS EC YST	n/a	Yes	Retroperitoneal lymph nodes	~6 years	Unknown

ChT, chemotherapy; EC, embryonal carcinoma; ENT, embryonic‐type neuroectodermal tumour; LGGT, low‐grade glial/ganglioglial tumour of germ cell origin; n/a, not applicable; NED, no evidence of disease; RMS, rhabdomyosarcoma; RPLND, retroperitoneal lymph node dissection; YST, yolk sac tumour.

* From the time of orchiectomy/the latest orchiectomy in patients with bilateral orchiectomy.

### RNA Extraction and microRNA Testing

LGGT areas were marked on glass slides by a genitourinary pathologist for manual dissection from 5 μm unstained FFPE sections. The microRNA testing pipeline used for analysis was previously described in detail.^23^ In brief, total RNA was extracted from LGGT, GCNIS‐derived primary TGCTs, and ENTs, using a commercial RNA/DNA purification kit (Norgen Biotek, Thorold, Canada). Quantification was performed in a NanoDrop™ Lite Spectrophotometer (Thermo Fisher Scientific, Waltham, Massachusetts, USA). An input of 100 ng of total RNA was used for reverse transcription using the TaqMan MicroRNA Reverse Transcription Kit (Applied Biosystems, Waltham, Massachusetts, USA). Quantitative real‐time Polymerase Chain Reaction (RT‐qPCR) was performed on the QuantStudio 12K Flex platform, using Xpert Fast Probe. TaqMan™ microRNA assays for hsa‐miR‐371‐3p (assay ID 002124), hsa‐miR‐372‐3p (assay ID 000560), and hsa‐miR‐373‐3p (assay ID 000561) were run. RNU48 (assay ID 001006) was used as the endogenous quality control. All reactions were run in triplicate. RNA extracted from the seminoma‐like cell line TCam‐2 was used as a positive control and plated in a dilution series to assess reaction efficiency. The negative controls included reactions with no template (NTC) and no cDNA.

### DNA Methylation Genome‐Wide Analysis

Twenty‐three samples were used for comparative genomic DNA methylation assessment, including 7 LGGTs profiled *de novo*, as well as 6 ENTs and 10 postpubertal‐type teratomas profiled as part of previous studies (^13^, ^17^). The areas of interest were manually dissected from FFPE tissues, and DNA was extracted for DNA methylation analysis as previously described (^13^, ^17^). LGGT samples were profiled using the Illumina EPICv2 array, and the remaining tumours had been assessed using the EPICv1 array. Raw IDATs generated from iScan were processed and analysed using the Bioconductor R package *Minfi*.^24^ To account for differences in array design, EPICv1 and EPICv2 samples were processed separately with the same method. Samples were filtered for quality using a mean detection *P*‐value threshold of <0.01, and probes were filtered with a mean detection *P*‐value threshold of <0.05. Then, probes were normalised using quantile normalisation and corrected for background signals. Probes overlapping SNPs were removed. Afterwards, data from different chips were combined by identifying overlapping probes on both arrays.

Considering that the EPICv2 array has a different design, we incorporated a new updated version of the EPICv2 manifest from Peters *et al*.^25^ choosing superior probes within replicates in terms of their sensitivity and accuracy. Beta values were calculated as the ratio of intensities between methylated and unmethylated alleles with an offset value of 100. Most variable probes were identified by calculating variance across all samples for each probe. The 10,000 most variable probes were used to generate the UMAP plot. Heatmaps were generated with the R package *ComplexHeatmap* ^26^ visualising the 1000 and 5000 most variable probes.

### Statistical Analysis

Micro RNA expression data was tabulated on GraphPad Prism 9 (GraphPad Software, Boston, USA). Expression values for each tumor type were described as median and interquartile range. MicroRNA results were considered as positive (Ct < 28), negative (Ct >35), and “grey zone”/clinically indeterminate (28 ≤ Ct ≤ 35), adapting the protocol and cutoffs described by Lafin *et al*.^27^ for miR‐371a‐3p testing in serum, as described previously.^17^, ^23^ Cases with “grey zone” results were plated on a second PCR run as recommended. Data was plotted as a 40‐Ct value for readability. The non‐parametric Mann–Whitney *U*‐test was used to compare microRNA levels between groups.

## Results

### Clinicopathological Features

**Figure 1:**
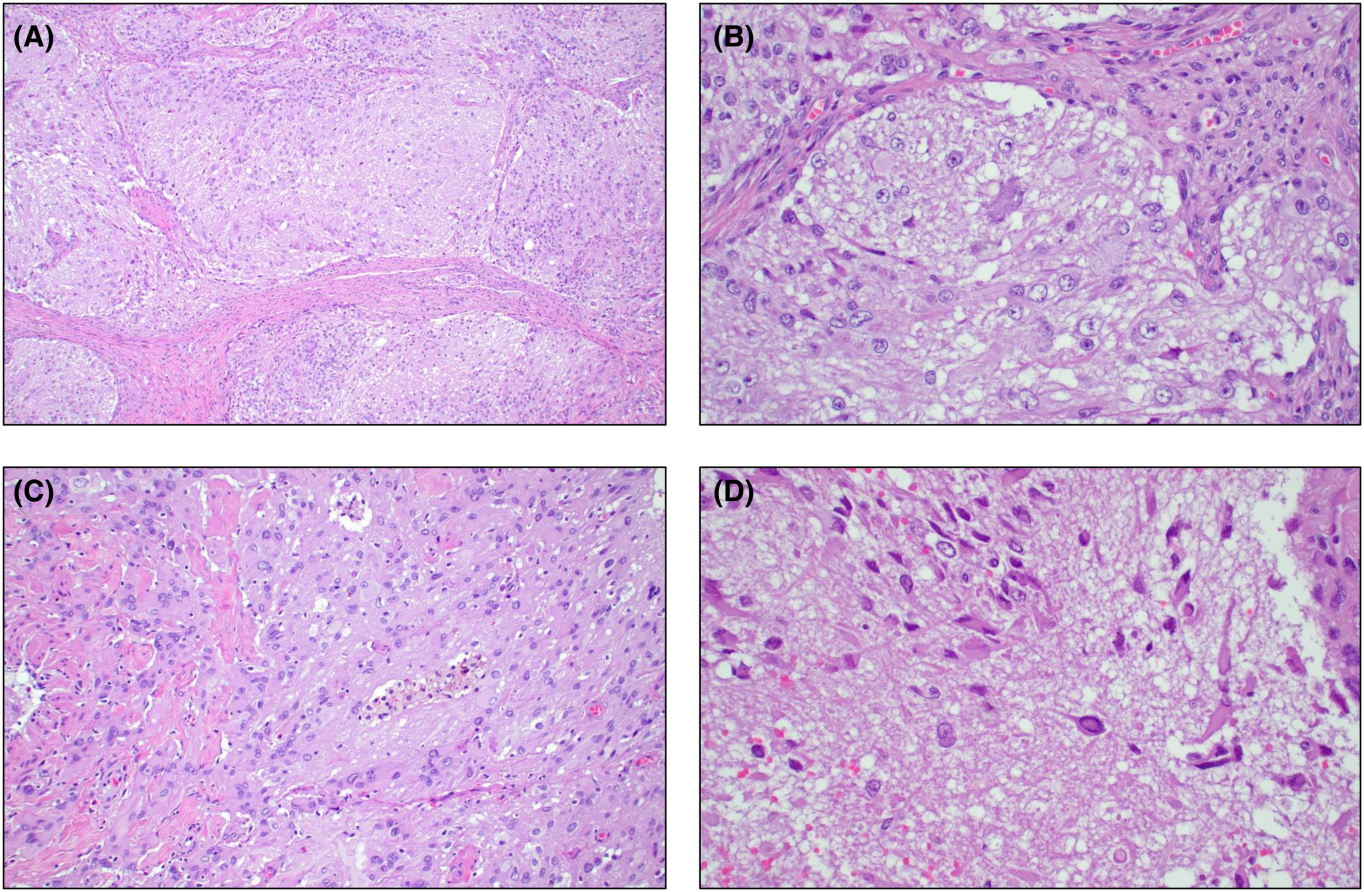
Histopathological features of the study cases. (**A, B**) Low grade glial tumours were composed of sheets and nests of cells with granular to fibrillary eosinophilic cytoplasm, round to oval nuclei, and centrally‐located nucleoli. Individual tumours showed areas with variable cellularity (best seen in A). Necrosis, brisk mitotic activity, and nuclear pleomorphism were not identified. (**C**) Some tumours contained aggregates of foamy and pigment‐laden macrophages. (**D**) Notice the prominent fibrillary neuropil‐like background and the presence of scattered developing ganglion cells, characterized by hyperchromatic nuclei and dense eosinophilic cytoplasm with delicate projections.

**Figure 2:**
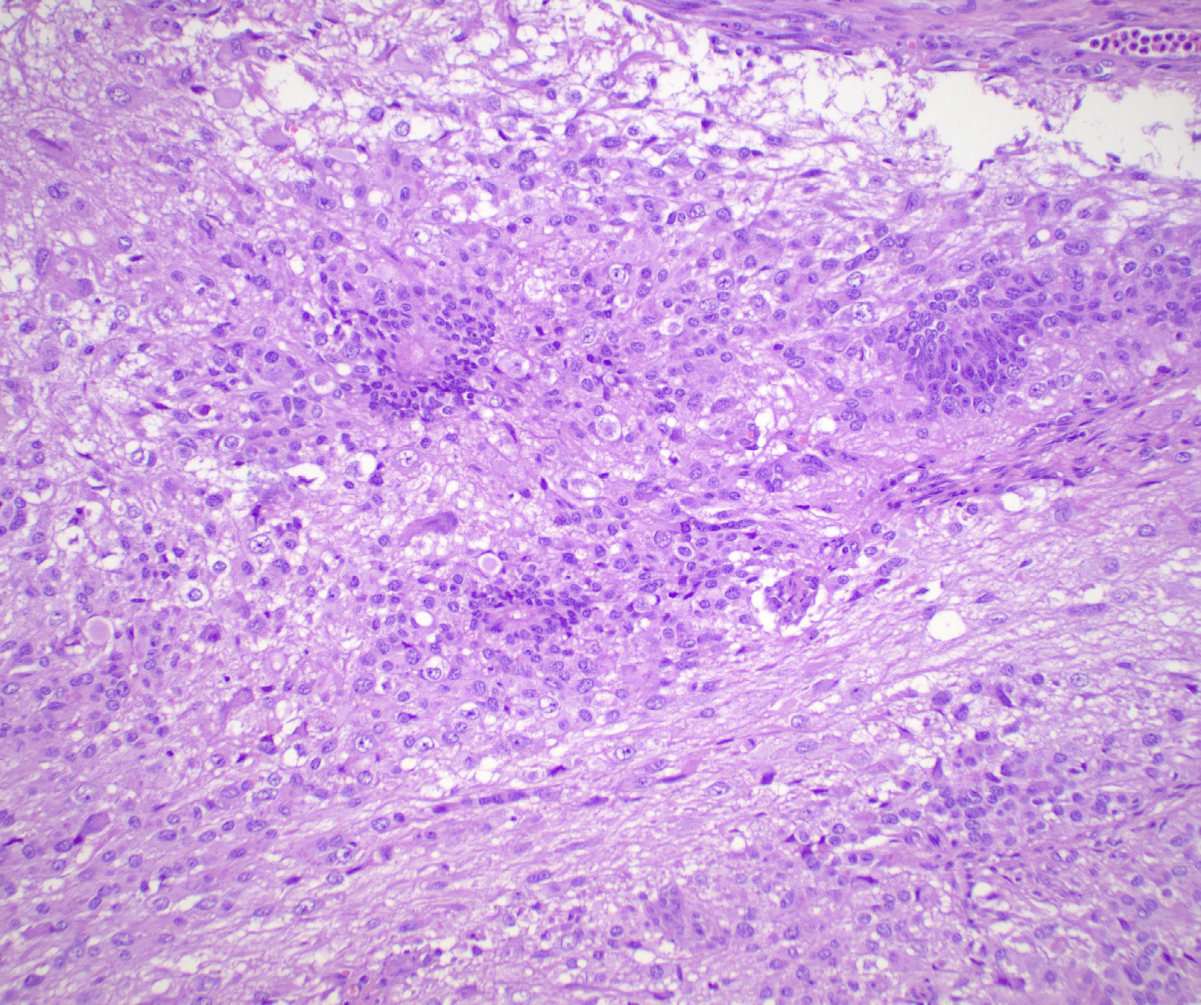
One tumour exhibited small scattered foci of embryonic type neuroectoderm, characterised clusters of primitive cells with scant cytoplasm and round hyperchromatic nuclei. Some of the neuroectodermal clusters are arranged in rosette‐like structures.

**Figure 3:**
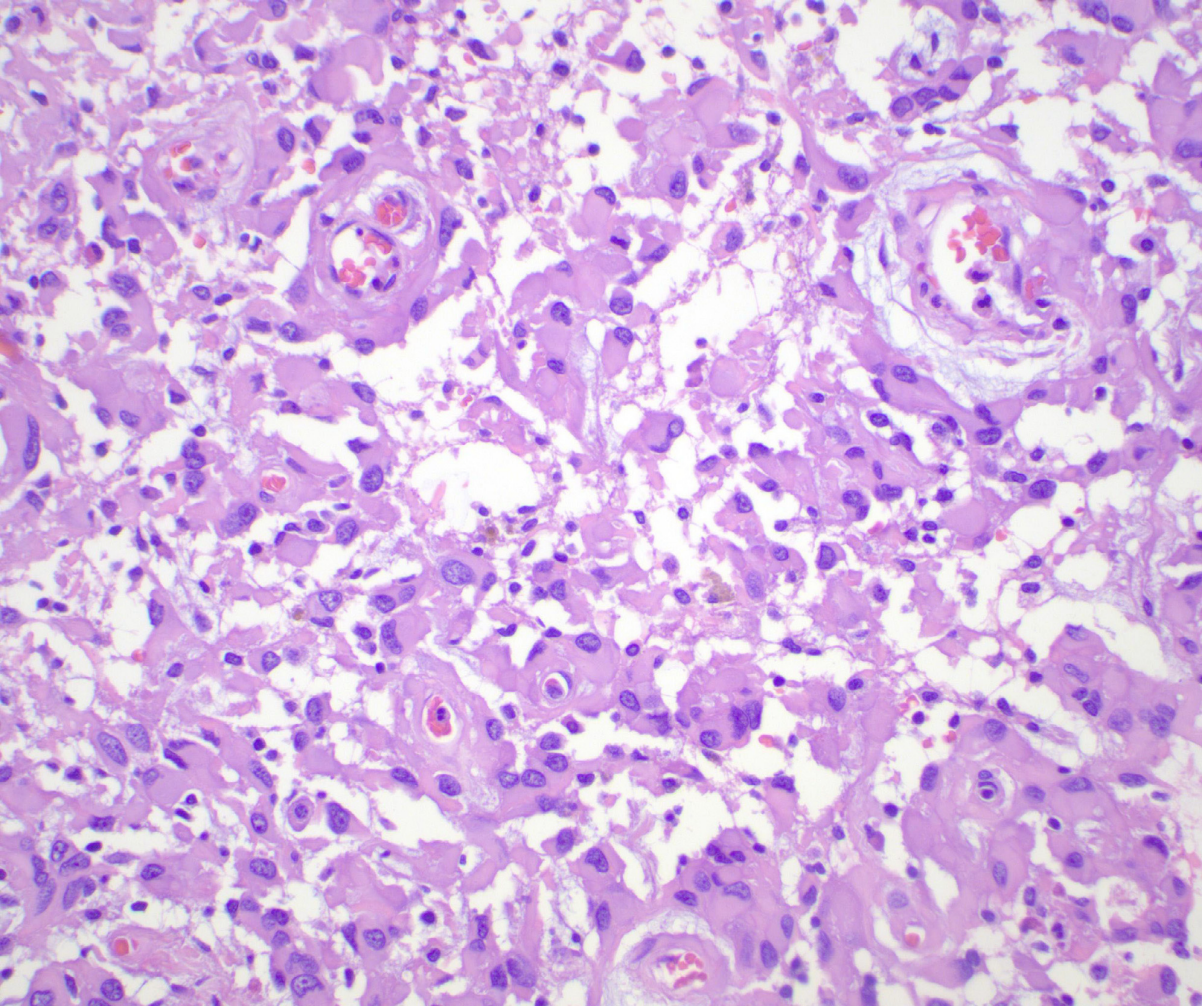
One tumour showed vague resemblance to gemistocytic astrocytomas, with sheets of large polygonal cells with abundant eosinophilic cytoplasm.

The clinicopathological features of the LGGTs included in the series are summarised in Table 1. The primary tumours were testicular in five patients and mediastinal/possibly mediastinal in the remaining two. LGGTs were present in recurrences after chemotherapy in six patients and in a primary mediastinal tumour in one patient. All tumours showed, by definition, low‐grade histologic features and formed a distinct nodule larger than 5 mm. Cellularity was highly variable, from low to relatively high; mitotic activity was low (<1 mitotic figure per 10 high‐power fields), and necrosis was not identified. Six tumours showed large atypical glial cells and scattered ganglion cells in a background of abundant neuropil (Figure 1). Microscopic foci of embryonic‐type neuroectoderm were identified in one of these six LGGTs (Figure 2), and three cases had a prior history of ENT. The remaining tumour was composed of sheets of large polygonal cells with abundant eosinophilic cytoplasm, resembling a gemistocytic astrocytoma, albeit with more pronounced nuclear atypia (Figure 3). Clinical follow‐up was limited; however, at least one patient (case 1) showed progressive disease after surgery and another patient (case 4) with a pure LGGT exhibited a high disease burden, requiring a multiorgan resection including pancreas, spleen, and kidney.

### MicroRNA Analysis

**Figure 4:**
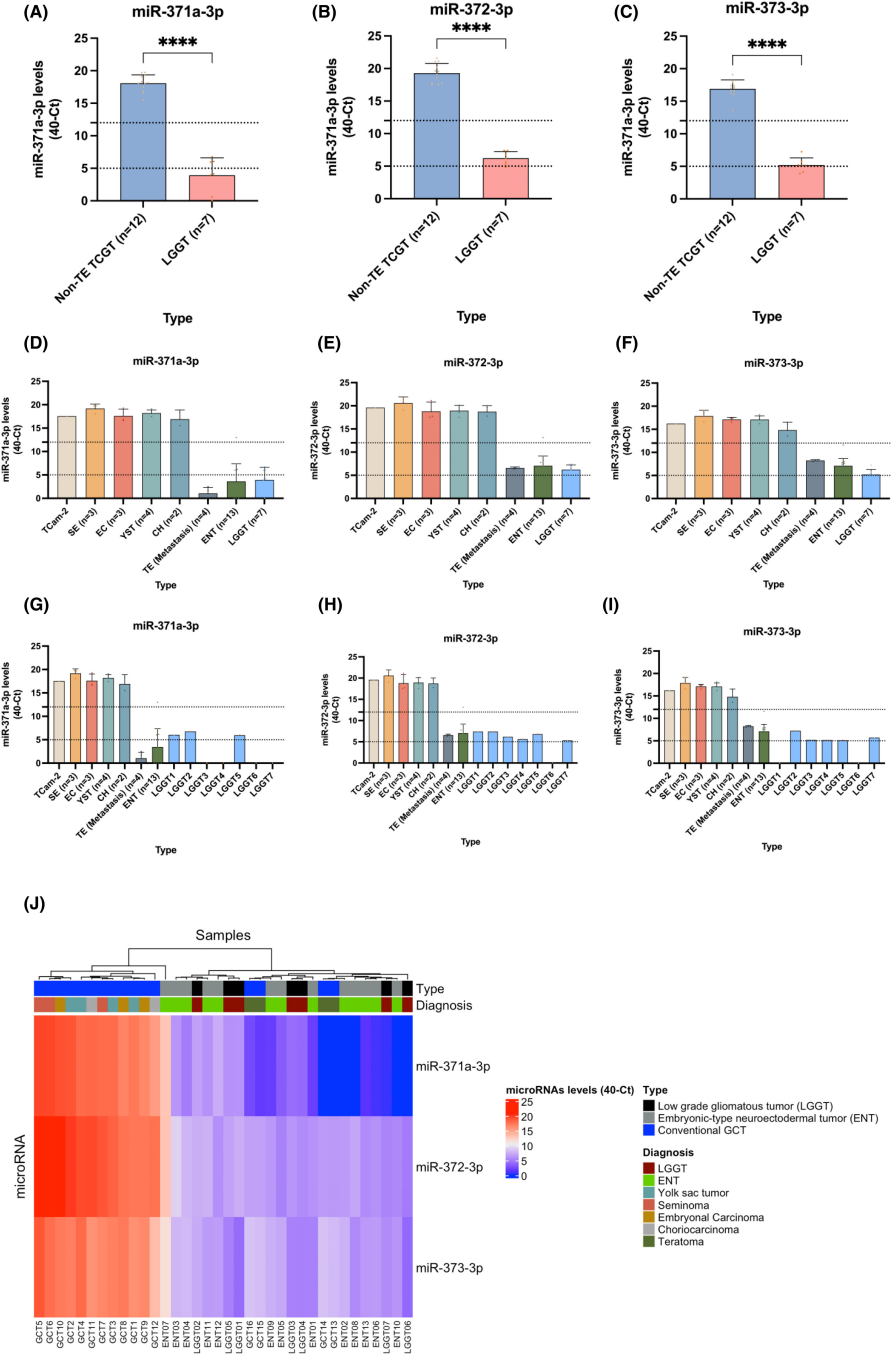
Levels of miR‐371a‐3p, miR‐372‐3p, and miR‐373‐3p in low grade glial/gliomatous tumours of germ cell origin (LGGT), embryonic‐type neuroectodermal tumour (ENT), and conventional testicular germ cell tumours (TGCTs). (**A–C**) Overall levels in LGGT versus non‐teratoma TGCT (considered as a single group). ****P < 0.0001. (**D–F**) Overall levels in LGGT compared with ENT and conventional histologic types of TGCT. (**G–I**) Levels of expression in individual LGGTs compared to median expression in ENT and conventional histologic types of TGCT. (**J**) Unsupervised clustering of samples based on microRNA 371~373 profiling, including LGGT, ENT, and conventional TGCTs (both teratoma and non‐teratoma). Results are plotted as 40‐Ct for readability. Median values are reported. Dashed lines define the categorisation of cases as positive (Ct < 28), grey zone (28 ≤ Ct ≤ 35), and negative (Ct > 35), as proposed by Lafin *et al*. The TCam‐2 seminoma‐like cell line is shown as a positive control. CH, choriocarcinoma; EC, embryonal carcinoma; ENT, embryonic type neuroectodermal tumour; LGGT, low‐grade glial/gliomatous tumour of germ cell origin; SE, seminoma; TE, teratoma; YST, yolk sac tumour.

Results of microRNA analyses are illustrated in Figure 4. Levels of all three microRNAs of the cluster were significantly higher in non‐teratoma TGCT than in LGGT (*P* < 0.0001 for all comparisons, Figure 4A–C). As expected, miR‐371a‐3p, miR‐372‐3p, and miR‐373‐3p were highly positive in seminoma, embryonal carcinoma, choriocarcinoma, and yolk sac tumour, and negative in teratomas (Figure 4D–F). RT‐qPCR for miR‐371a‐3p, the most specific member of the cluster, showed that four LGGTs were negative, whereas the remaining three showed levels within the “grey zone.” The latter included two LGGTs associated with teratoma and one LGGT associated with teratoma and yolk sac tumour. Results for miR‐372‐3p and miR‐373‐3p followed the same general pattern (Figure 4G–I). Unsupervised clustering based on microRNA 371~373 expression profiles showed two clusters: one composed of LGGTs, ENTs, and teratomas (intermingled with each other), and another including the non‐teratoma TGCTs (Figure 4J).

### DNA Methylation Profiling

**Figure 5:**
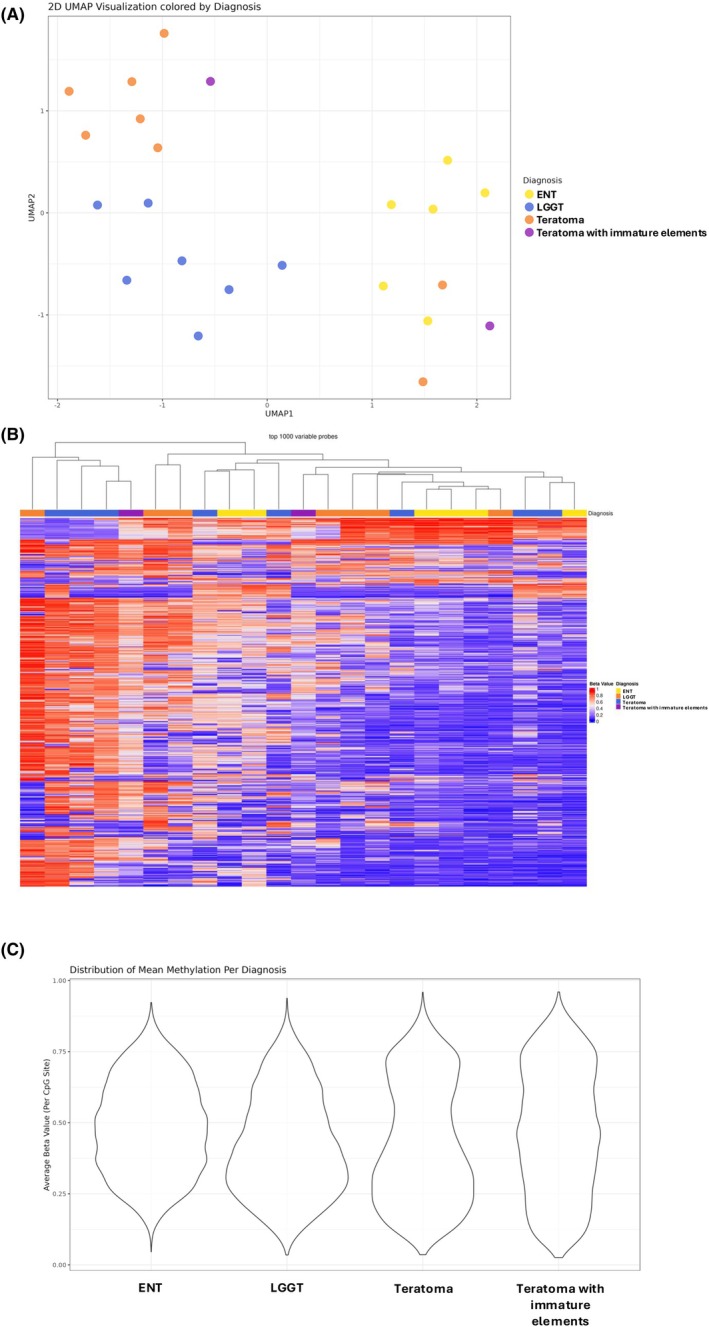
Genomic DNA methylation profiling. (**A**) 2D UMAP projection visualising the clustering of samples. Each point represents an individual sample, and colours indicate their diagnosis: embryonic‐type neuroectodermal tumour (ENT; yellow), low grade glial tumour of germ cell origin (LGGT; blue), teratoma (orange), and teratoma with immature elements (purple). (**B**) Heatmap showign the results of unsupervised hierarchical clustering according to the top 1000 most variable probes. Each row represents a probe, and each column represents a sample. Beta values range from 0 to 1, with >0.8 as hypermethylated (red) and <0.2 as hypomethylated (blue). Diagnoses are annotated above the heatmap and colour‐coded: ENT (yellow), LGGT (orange), teratoma (blue), and teratoma with immature elements (purple). (**C**) Violin plots showing the distribution of average beta values per CpG site for the top 10,000 most variable probes in each histologic type (same probes used for UMAP dimensionality reduction).

2D UMAP projection of investigated tumour samples showed no definitive clustering according to histology; however, LGGTs appeared to form a somewhat loose cluster (Figure 5A, lower left). The heatmap representation of unsupervised clustering based on the top 1,000 most variable probes showed the absence of clustering according to histology, with the major subclusters comprising a mixture of teratoma, ENT, and LGGTs (Figure 5B). Genome‐wide methylation assessment using violin plots generated with the top 10,000 most variable probes demonstrated distinct profiles corresponding to each histologic type, with higher frequency/abundance of sites with intermediate methylation levels in LGGTs and ENTs compared to “mature” teratomas (i.e., teratomas lacking immature elements, Figure 5C). Of note, the profile of distribution of beta values in LGGT had an appearance that was “intermediate” between ENT and “mature” teratoma (Figure 5C).

## Discussion

So‐called “somatic‐type” malignancies (SMs) are neoplasms of germ cell origin that resemble a true somatic tumour and occur in ~2.5%‐8% of TGCTs overall.^28^ Although they are seen more commonly after chemotherapy, they are also recognized in a small subset of primary germ cell tumours.^9^ Post‐chemotherapy SMs are associated with a poor prognosis, with a 5‐year cancer‐specific survival of 64% and overall 5‐year survival of 35%,^10^, ^29^ due in part to their resistance to conventional systemic treatment.

**Figure 6:**
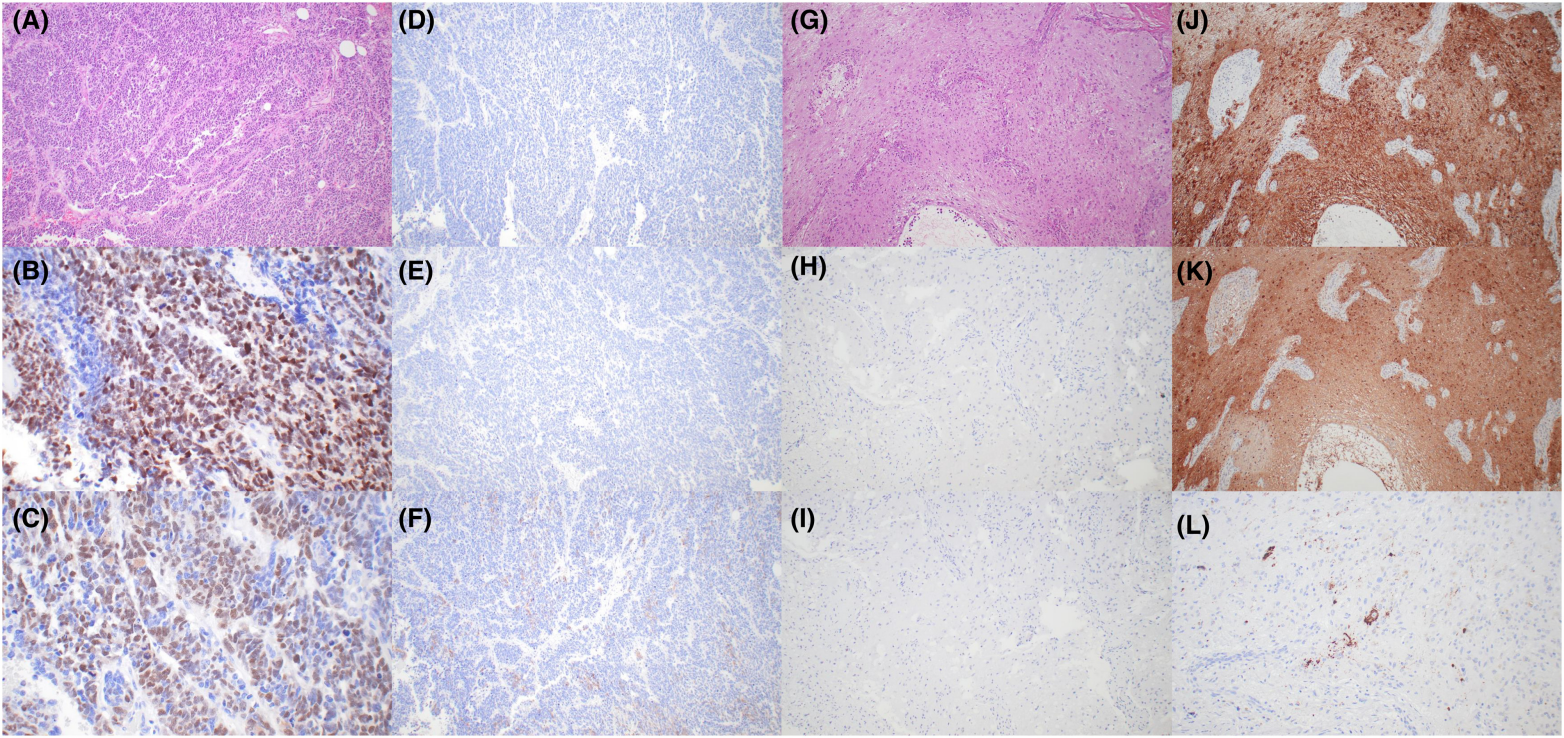
Immunophenotype of embryonic‐type neuroectodermal tumour (ENT) and low grade glial tumours of germ cell origin (LGGT). (**A**) ENT (**A–F**), with typical morphology (**A**) and expression of SOX11 (**B**) and SOX2 (**C**). The tumour is negative for GFAP (**D**) and S100 (**E**), whereas synaptophysin is expressed in scattered foci (**F**). LGGT (**G–L**), with typical morphology (**G**) and absence of expression of SOX11 (**H**) and SOX2 (**I**). The tumour is diffusely positive for GFAP (**J**) and S100 (**K**). Synaptophysin highlights scattered ganglion cells (**L**).

The current WHO classification of urinary and male genital tumours (2022) arbitrarily defines SMs as discrete foci with somatic‐type histology measuring at least 5 mm.^12^ SMs encompass neoplasms with varied histology, including primitive embryonic‐type tumours (such as ENT or nephroblastoma), sarcomas, carcinomas, hematolymphoid tumours, and glial/neuroglial tumours.^6^, ^7^, ^8^, ^13^ The latter are rare (1 out of 124 cases of SM^8^) and occur mostly after chemotherapy. Glial/neuroglial tumours of germ cell origin span a spectrum, including low‐grade and high‐grade lesions according to the criteria used to assess CNS tumours. These glial/neuroglial tumors have been likened to low‐grade astrocytomas and gliomas, anaplastic astrocytomas, glioblastomas, gangliogliomas, and gliosarcomas of the nervous system.^19^ Many exhibit intratumoral grade variability,^30^ and they consistently lack the genetic drivers of their true somatic counterparts, including *BRAF*, *ATRX*, and *IDH* alterations.^19^ ENT, a SM characterized by proliferation of primitive (embryonic‐type) neuroectoderm and expression of transcription factors of pluripotential neuroglial precursors such as NKX2.2, SOX11, and SOX2, are frequently intermingled with areas of mature glial/ganglioglial differentiation.^20^, ^30^, ^31^ Particularly interesting is a group of tumours with pure mature glial or ganglioglial histology that resemble low‐grade glial and ganglioglial/neuroglial neoplasms of the nervous system (termed LGGTs in this manuscript). Although these neoplasms are often recognisable by morphology, immunohistochemistry can occasionally be helpful or confirmatory (the most relevant characteristics of the immunoprofile of LGGTs and ENTs are summarised in Table 2 and representative illustrations are shown in Figure 6). Based on clinicopathologic observations, we hypothesise that LGGTs represent a phenomenon of maturation of ENT, either induced by therapy or spontaneous, akin to the maturation of conventional histologic subtypes of GCT to teratoma. However, the origin of LGGTs and their biologic relatedness to other histologic subtypes of GCNIS‐derived GCTs remain poorly understood.

**Table 2:** Immunohistochemistry overview (frequent patterns) of LGGT and ENT arising in GCTs based on literature search

Marker	LGGT	ENT
SOX11	−/+ (focal, in immature cells)	+++
SOX2	−/+ (focal, in immature cells)	+++
Synaptophysin	+ (highlights ganglion cells)	−/++ (typically highlights scattered “mature” foci)
Chromogranin	+ (highlights ganglion cells)	−/++ (highlights small “mature” foci)
S100	+++ (diffuse)	−/+ (focal)
GFAP	+++ (diffuse)	−/+ (focal)

TGCTs are characterised by epigenetic traits (including microRNA and DNA methylation profiles) that reflect the developmental potential of their purported cell of origin.^32^, ^33^ While the genomic alterations of paired SM and conventional components of individual GCTs are largely shared (i.e., present in both components), their microRNA expression and DNA methylation profiles show noticeable differences, suggesting that epigenetic events may underlie the phenotypic switch.^17^ MicroRNAs are short (20–24 nucleotide‐long) segments of RNA that do not code for proteins and function as post‐transcriptional regulators of gene expression, being implicated in several disease processes, including cancer.^34^ MicroRNAs represent excellent circulating biomarkers because they are stable in body fluids and can be detected with a low‐cost, readily accessible methodology (RT‐qPCR).^35^ MicroRNAs of the 371~373 cluster, and miR‐371a‐3p in particular, have proven their worth as circulating biomarkers of GCNIS‐derived TGCTs, being >90% sensitive and specific for non‐teratoma.^36^, ^37^, ^38^ Under the assumption that mature components of teratoma are “terminally differentiated” and cannot revert to a pluripotent phenotype, these embryonic microRNAs can be used to assess the relatedness of rare phenotypes of TGCT to teratoma and non‐teratoma. For instance, rare cystic trophoblastic tumours of germ cell origin are negative for miR‐371a‐3p, suggesting that they represent “maturation” of choriocarcinoma, being biologically equivalent to teratoma.^39^ In contrast, the expression of microRNAs of the 371~373 cluster in a subset of SMs and in sarcomatoid yolk sac tumours suggests that their biologic and developmental potential is more similar to that of yolk sac tumour or other pluripotential subtypes of non‐teratoma than to that of teratoma.^17^ In the present study, LGGTs showed an absence of significant microRNA 371~373 expression, supporting that their phenotype is “mature” and similar to that of teratoma. The 3 LGGTs with results within the “grey zone” could represent maturing neoplasms with an intermediate phenotype. In support of this concept, at least one LGGT contained small microscopic foci of embryonic‐type neuroectoderm.

The genomic DNA methylation profile of TGCTs is also quite variable among histologic subtypes of TGCTs, from globally hypomethylated in some seminomas to hypermethylated in specific regions in non‐seminomas.^22^ In general, increased global DNA methylation parallels the process of maturation towards teratoma, with the highest overall levels of methylation seen in the latter histologic subtype.^40^ Also, paired analyses of cisplatin‐sensitive and cisplatin‐resistant TGCTs has shown increased genome‐wide DNA methylation in resistant neoplasms.^41^ In the present study, comparative analyses based on the top differentially methylated probes showed that LGGTs, ENTs, and teratomas did not form discrete clusters, possibly reflecting that they represent a continuum of maturation. Genome‐wide analyses using violin plots generated with the top 10,000 differentially methylated probes demonstrated a distinct methylation profile in each tumour type. Specifically, ENT and LGGT showed a higher abundance of regions with intermediate methylation than teratoma, whereas teratoma exhibited a higher abundance of hypermethylated regions. Of note, the genome‐wide DNA methylation profile of LGGTs was more similar to that of ENT than to that of teratoma. Although the number of samples is somewhat limited, these data suggest that LGGTs are part of the spectrum of ENT, likely representing “maturation” of the latter. This is also supported by the presence of ENT in a prior sample in three cases and small foci of embryonic neuroectoderm within the LGGT in one case. The differences in the methylation profiles of teratoma and LGGT are not surprising considering their histologic differences. Specifically, while the LGGTs that were analysed exhibited pure glial or neuroglial/ganglioglial histology, teratomas used as comparators were more heterogeneous, including different combinations of epithelial and mesenchymal elements.

In summary, our findings suggest that post‐chemotherapy LGGTs result from differentiation of ENT components and are, most likely, akin to teratoma from a biological perspective. In this scenario, chemotherapy may induce the maturation, or (perhaps most likely) select existing subclones with a “mature” phenotype that give rise to LGGTs. Like teratoma, LGGTs are chemoresistant but relatively indolent if completely resected; nonetheless, further studies are needed to assess their clinical behaviour in greater detail. Complete resection with negative surgical margins is desirable, if attainable, until this entity is better understood. From a practical perspective, our results show that LGGTs do not significantly express microRNAs of the 371~373 cluster and will therefore be overlooked by assays for circulating miR‐371a‐3p.

## Author contributions

Concept design and coordination: JL and AMA. Writing—drafting manuscript: JL, NTT. Drafting of table and clinical information collection: MH. Methodology: JL, NTT, FFP, CJ, YY, MS, MI, AMA. Provision of cases: JL, RH, MI, TU, AMA. Writing—critical revision of draft: TU, AMA. All authors read and approved the final manuscript.

## Conflict of interests

The authors declare that they have no conflicts of interest.

## Data Availability

All data produced in this work are presented in the manuscript and corresponding files. The raw data generated during this research are available from the corresponding author upon reasonable request.
